# Control of malaria and other vector-borne protozoan diseases in the tropics: enduring challenges despite considerable progress and achievements

**DOI:** 10.1186/2049-9957-3-1

**Published:** 2014-01-08

**Authors:** Denis Zofou, Raymond B Nyasa, Dickson S Nsagha, Fidele Ntie-Kang, Henry D Meriki, Jules Clement N Assob, Victor Kuete

**Affiliations:** 1 Biotechnology Unit, Faculty of Science, University of Buea, P.O. Box 63, Buea, South West Region, Cameroon; 2 Department of Public Health and Hygiene, Faculty of Health Sciences, University of Buea, Buea, Cameroon; 3 CEPAMOQ, Faculty of Science, University of Douala, P.O. Box 8580, Douala, Cameroon; 4 Chemical and Bioactivity Information Centre, Department of Chemistry, University of Buea, P. O. Box 63, Buea, Cameroon; 5 Department of Biomedical Sciences, Faculty of Health Sciences, University of Buea, Buea, Cameroon; 6 Department of Biochemistry, University of Dschang, P.O. Box 67, Dschang, Cameroon

**Keywords:** Malaria, Vector-borne protozoan diseases, African trypanosomiasis, Chagas disease, Leishmaniasis, Vector control, Chemotherapy, Vaccine development

## Abstract

Vector-borne protozoan diseases represent a serious public health challenge, especially in the tropics where poverty together with vector-favorable climates are the aggravating factors. Each of the various strategies currently employed to face these scourges is seriously inadequate. Despite enormous efforts, vaccines—which represent the ideal weapon against these parasitic diseases—are yet to be sufficiently developed and implemented. Chemotherapy and vector control are therefore the sole effective attempts to minimize the disease burden. Nowadays, both strategies are also highly challenged by the phenomenon of drug and insecticide resistance, which affects virtually all interventions currently used. The recently growing support from international organizations and governments of some endemic countries is warmly welcome, and should be optimally exploited in the various approaches to drug and insecticide research and development to overcome the burden of these prevalent diseases, especially malaria, leishmaniasis, Human African Trypanosomiasis (HAT), and Chagas disease.

## Multilingual abstracts

Please see Additional file 1 for translations of the abstract into the six official working languages of the United Nations.

## Background

Major vector-borne protozoans of public health concern in the tropics include Sporozoa, Rhizopoda, Ciliates, and Flagellates. Diseases caused by Plasmodia (malaria), and three major trypanosomatid diseases [leishmaniasis, African Human Trypanosomiasis (HAT) and Chagas disease] represent a major public health concern in the tropics. Malaria, for example, is the world’s most important parasitic disease especially when *Plasmodium falciparum* is the causative agent. The disease is endemic in more than 100 developing countries where it accounts for about 40 to 45 million DALYs (Disability-Adjusted Life Years). The malaria burden, however, is slightly decreasing, and it is unevenly distributed worldwide: 35 countries, among which 30 in are Sub-Saharan Africa and five are in Asia, account for 98% of global malaria deaths [1,2]. Trypanosomatid diseases are classified as “Tropical Neglected Diseases” by the World Health Organization (WHO) because of the lack of attention—both at the community, national, and international levels—these infections are paid, despite their heavy burdens, particularly in the tropics [3,4].

The present review discusses and analyzes the major strategies currently employed in an effort to minimize the burden of these diseases, and the major progress and achievements resulting from international as well as local efforts. Valued reports are comprehensibly documented on prevention methods (vector control and vaccines), management tools (chemotherapy, global and regional coordination of control strategies), and the international and local support to research and development targeting the selected diseases. The information was retrieved using the major keywords presented in this review, and the duplicated data eliminated, with priority given to the earlier sources of similar information. In brief, data was collected from 145 articles (appeared in 68 peer-reviewed journals), four textbook chapters, 17 reports by international organizations, and eight web-published fact sheets, published between 1945 and 2013. The information gathered was analyzed and discussed grouped into the major thematic subjects as presented in the result section below.

## Review and discussion

### Progress and challenges in prevention methods

The prevention of vector-borne diseases often consists of blocking the transmission from one person to another through vectors, and immunization of individuals against the disease by vaccination or chemoprophylaxis. The first strategy is permanently challenged by several limitations whereas the vaccine enterprise for parasitic infections is yet to bear expected fruits. Chemoprophylactic methods will be discussed under the section on chemotherapy.

#### Vector control

**Figure 1:**
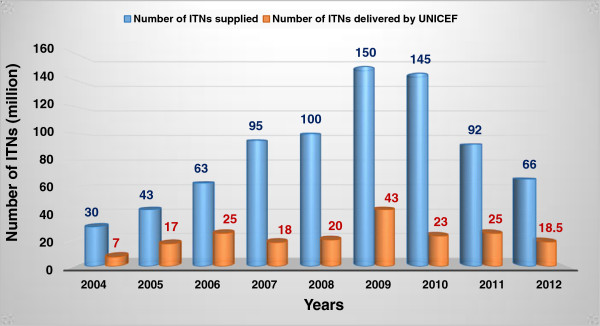
**Efforts of the international community towards vector control of protozoan diseases: 2004–2012.** Source: WHO [2], PATH [89]; UNICEF Supply Reports for 2010, 2011 & 2012, available at http://www.unicef.org/supply/index_68730.html; ITNs: insecticide-treated bednets.

About 500 different species of *Anopheles* exist, up to 60 of which transmit the disease. The most common species are *Anopheles gambiae*, *A. arabiensis*, *A. obscursus*, *A. guadrimacutis*, *A. nili*, and *A. moucheti*[5]. Therefore, the vector distribution determines the malaria distribution and endemicity. For example, in the USA and Europe where climatic factors are not favorable to *Anopheles*, malaria is very rare or absent. Vector control is usually achieved using environmental management, biological methods, or insecticides which are either directly sprayed indoors or applied to bed nets (see Table 1) [2,6-8]. Treatment directed towards mosquito larvae consists of destroying larvae nests using any of the following methods: *i) Environmental management methods* comprising of filling breeding sites, lining water sources and canals, physical wetland drainage, biological wetland drainage, impoundment planning, deepening and narrowing of old drains, vegetation manipulation, synchronized cropping and intermittent irrigation, larvivorous fish introduction, and saltwater flooding; ii) *Larvicidal agents:* bacterial larvicides, methoprene, temephos, and molecular films and oils [5]. It is reported that from 2008 to 2010, a cumulative total of 254 million Insecticide Treated Bed Nets (ITNs) were distributed in Sub-Saharan Africa to cover 66% of the 765 million persons at risk in the continent (see Figure 1). However, in order to be effective, bed nets should be regularly re-treated with insecticides, and there is also the serious problem of compliance related to sociocultural considerations in certain communities [9-11].

**Table 1:** Types of interventions in vector control and their limitations

**Control methods**	**Advantages**	**Limitations**
Environmental hygiene (eliminates vector’s nests, etc.)	Efficacy > 50%	Necessitates community-guided actions
Indoor Residual Spraying (IRS)	Efficacy 60%	Harmful effects of residues, high cost, resistant strains of mosquitoes
Intermittent Preventive Treatment (IPT)	Efficacy 56%	May enhance drug resistance, use restricted for pregnant women and children < 5 years
Insecticide treated mosquito bed nets	Efficacy 50%	High cost, poor adherence of rural communities, harmful effects of residues, resistance
Biological control	No direct harmful effects on humans	Cost, genetic risks

**Source**: Curtis [7], Lengeler [8], Parise [9], Morel et al. [10].

The major well characterized active ingredients of all WHO-recommended products for mosquito bed nets and Indoor Residual Spraying (IRS) come from four classes of insecticide: pyrethroids, organochlorines (dichlorodiphenyltrichloroethane, DDT), organophosphates, and carbamates. Among these, pyrethroids are by far the best class, both in terms of safety and effectiveness. However, the emergence and rapid spread of mosquito strain presenting insecticide resistance has become a major concern, as the phenomenon is now reported in more than 60% of malaria-endemic countries, with all major vector species and all classes of insecticides affected [12]. Four major types of insecticide resistance exist in *Anopheles*, namely target-site resistance, metabolic resistance, cuticular resistance, and behavioral resistance. Target-site resistance is caused by a gene mutation affecting ion channels, leading to evasion of the target of the insecticide molecule. Metabolic resistance occurs when increased levels or modified activities of an enzyme system cause a premature deactivation of the insecticide before it reaches its target in the mosquito. The major enzyme systems often concerned in metabolic resistance by premature deactivation are: esterases, monooxygenases, and glutathione S-transferases. In cuticular resistance, a modification in the composition or structure of the mosquito’s cuticle hinders the permeability of the insecticide, leading to a poor absorption and reduced efficiency. Such mechanism has been observed with pyrethroid in *Anopheles funestus* due to an abnormal thickening of the insect’s cuticle. Probably following continuous exposure to a particular insecticide, mosquitoes may modify their feeding and breeding behaviors so as to avoid the lethal effects of the insecticide. This type of resistance is termed behavioral resistance [12]. It is noteworthy underlining that the same mosquito can display more than one resistance type towards a single or several insecticides and this complex situation is termed cross-resistance. In order to address the issue of insecticide resistance, WHO [12] advised four major strategies: i) *Rotations of insecticides*, implying the use of two or more insecticides with different mechanisms of action from one year to the next; ii) *Combination of interventions*, using two or more molecules with different target sites in the same house so as to expose the mosquitoes simultaneously to different types of insecticides; iii) *Mosaic spraying*, characterized by the use of one insecticide in a geographic area and a different molecule in neighboring areas; like combination of interventions, the mosquitoes are exposed to more than one insecticide type; iv) *Mixtures*, in which two or more insecticides classes are mixed to make a single product or formulation. However, despite these strategies being well justified and rational, it remains difficult to predict how far these new chemical-based approaches could succeed in eliminating malaria and other vector-borne infections in the tropics. Each of these strategies has both advantages and limitations, and all necessitate concerted efforts at both national and regional levels, which are not always achievable.

Biological methods have recently attracted growing attention, certainly due to their relatively low cost and their assumed safety, compared to insecticides. In this regard, several options have been envisaged, including the use of refractory mosquitoes and paratransgenic organisms. In fact, most species of mosquitoes do not transmit malaria, and even among species that do, many individuals seem incapable of transmitting the disease [13]. The existence of such refractory mosquitoes represents a hope that the genes that permit malarial infections in mosquitoes can be identified and knocked out, generating harmless transgenic mosquitoes. Spreading genetically-modified mosquitoes will eventually replace the natural malaria-transmitting mosquito populations, and halt malaria transmission. A variety of methods for engineering refractory mosquitoes are currently being studied, with promising results in rodent malaria. Fang et al. [14] described the use of genetic manipulation techniques to insert multiple anti-malaria effector genes into the entomopathogenic fungus Metarhizium anisopliae. When such a modified fungus was used to infect *Anopheles* mosquitoes, it could express efficient anti-malaria effector molecules in the mosquito hemolymph. By co-expressing several effector molecules simultaneously, the authors observed a drastic reduction in sporozoite levels in the mosquito salivary glands reaching up to 98%. These findings suggest hope in the exploration of recombinant entomopathogenic fungi as a strategy to control malaria. Paratransgenesis has equally been shown to be highly promising in other vector-borne diseases, notably the dengue mosquito [15]. Further investigation into this innovative approach are therefore, highly encouraged.

### The concept of integrated vector management

In order to minimize some of the challenges persisting in vector control of the selected diseases and optimize interventions, in 2004, the WHO adopted a new strategy termed Integrated Vector Management (IVM) [16]. This is “a rational decision-making process targeting the global targets set for vector-borne disease control, by making vector control more efficient, cost effective, ecologically sound and sustainable”, based on five key elements: 1) evidence-based decision making, 2) integrated approaches 3), collaboration within the health sector and with other sectors, 4) advocacy, social mobilization, and legislation, and 5) capacity-building [16,17]. The WHO equally strongly recommends that other important sectors such as agriculture, environment, mining, industry, public works, local government, and housing, incorporate IVM and vector control into their own programs to help prevent vector proliferation and disease transmission due to their activities. The strategy could target several vectors simultaneously. Such an approach—if rigorously implemented—is very likely to yield great achievements. Success stories of IVM have been reported in several countries in Africa, including Tanzania, Nigeria, Zambia, and Sudan [18-21]. However, this success is tributary of good leadership and managerial governance from decision makers, and required an acceptable level of socioeconomic understanding and education of local populations. Unfortunately, such conditions are still to be met in most endemic tropical countries where the rate of poverty remains high. This situation may justify the slow progress, which continues to be observed with the implementation of IVM in Africa [17]. Therefore, IVM strategies need both technical and financial support from the international community in order to bear the expected fruits.

#### Progress and challenges in vaccine development for malaria and other protozoan diseases

##### Malaria

Malaria vaccine projects today target any of the three phases of the parasite lifecycle in humans. A vaccine preparation targeting any of the steps of the parasite lifecycle could either be envisaged from an attenuated whole organism, or could be made up of sub-unit antigens [22]. Whole-parasite malaria vaccine was thought feasible since the trials conducted in the 1960s and 1970s that showed sterile, long-lasting protection in mice and humans after vaccination with radiation-attenuated sporozoites. Immunization by mosquito bite with whole Pf sporozoites could consistently induce greater than 90% of protection against the infection, with the protection being sustained for at least 10–28 months. But these findings were hardly reproducible [23]. Recently, Professor Hoffman’s team at the Vaccine Research Center, National Institute of Allergy and Infectious Diseases (NAID), USA, has steadily worked on this approach with the vaccine candidate named PfSPZ, which has now completed the Phase I trial involving 40 voluntary adults. These studies confirmed a dose-dependent immunological threshold for establishing high-level protection against malaria that can be achieved by four doses of this vaccine. It is hoped that the following clinical trials, soon to start in several countries (Tanzania, USA, Mali, Germany, and Equatorial Guinea), would confirm the efficacy and suitability of this vaccine candidate [24]. The major challenge with the use of the whole-organism approach in malaria vaccine, if successful, would be that it requires huge quantities of biological material to meet the high need especially for endemic regions, which may be unfeasible.

As opposed to whole-organism vaccines, subunit vaccines are made up of a single parasite antigen or a combination of several antigens which have been shown to be vitally involved in infection mechanism. Such vaccine candidates achieved lower efficacy than whole-parasite vaccines, but are simpler and represent the class of vaccine candidates that have gone further in the development pipeline. One of these subunit vaccines named RTS,S is a hybrid molecule constructed by fusing the hepatitis B surface antigen (Haig) to the C-terminal half of the *P. falciparum* CSP (amino acid residue 207–395) and co-expressed with unfused HBsAg. The fusion protein is incorporated with an adjuvant termed AS02, based on monophosphoryl lipid A and QS-21 [25,26]. Clinical trial on RTS,S began in the USA in 1992 and in Africa in 1998, and has been gradually stimulated by promising results. Today, RTS,S is the first malaria vaccine candidate to reach the large-scale Phase III clinical testing, which is typically one of the last steps before regulatory approval. This phase started in May 2009 in Tanzania, one of the seven Sub-Saharan African countries hosting the 11 trial sites of the study. Enrolment of participants was completed in January 2011, with a total of 15,461 confirmed participants, including 6,538 infants aged 6–12 weeks, and 8,923 children aged 5–17 months. If the final results of this phase are once more conclusive, the WHO has indicated that a policy recommendation for RTS,S would then be possible, as early as 2015 [27,28]. However, the primary results of this Phase III trial are not as encouraging as the ones of the previous phases. The vaccine candidate reduced severe malaria by about 36.6% in the younger children aged 6–12 weeks only, and approximately 50% in the older age group (5–17 months) [28-30]. Moreover, how soon this vaccine (if approved for use) would be available and affordable to poor populations of remote areas in Sub-Saharan Africa remain questionable. In general, some of the well-recognized factors that have hindered the development of an effective vaccine for malaria include genetic complexity of the malaria parasite (genetic variation across stages), lack of understanding of the host mediators of natural immunity, lack of appropriate assays and surrogates for vaccine safety and efficacy, limited number of antigens being pursued as vaccine candidates, few funding programs to support the vaccines research enterprise, and limited number of immune enhancing adjuvants and vaccine delivery platforms available for use in humans among others [31-33].

##### Chagas disease

Advances and challenges towards a vaccine against Chagas disease have been extensively reviewed [34]. Briefly, a wide range of formulations has been tested, including whole parasites, purified or recombinant proteins, viral vectors, and DNA vaccines. Live attenuated *T. cruzi* whole organism was shown to confer partial immunity, with significant decreased parasitemia, and lower disease manifestation, especially heart disease. Similar results were observed with live *T. rangeli*, an inoffensive form of the parasite in humans. In addition to the effects noticed with attenuated *T. cruzi*, *T. rangeli* improved survival considerably. However, total immunity was never observed with the whole parasite vaccine. Another major limitation of this type of vaccine formulation (like in the case of malaria) is that it requires a large amount of biological material, which is quite challenging to generate, in order to immunize the number of populations in need. In attempt to overcome these limitations, several recombinant proteins have been prepared and tested for their potential to protect against Chagas disease. The most prominent ones are rASP-2 combined with Alum or CpG ODN, rTS (trans-sialidase) in combination with CpG ODN, rCruzipain + CpG ODN, and rGP82 + CpG ODN. All stimulated cytokines production lead either to decreased parasitemia and decreased burden, or decreased inflammation, with variable effects on the survival [34]. Recombinant virus vectors have also been designed and tested. The most prominent examples are the adenoviruses expressing TSSA CD8 epitope, TS and ASP-2, and the Sendai virus expressing ASP-2. Both are likely to provide x alternatives for immune protection. One of the major challenges limiting these efforts is the design and feasibility of clinical trials, given that the chronic form of the disease usually takes several years to develop, and concerns only 20% to 40% of infected patients. It is therefore very challenging to rationally follow up and draw conclusions from a clinical trial on Chagas disease vaccine development.

##### Human African trypanosomiasis

The initial vaccine targets of trypanosomiasis were the variable surface glycoproteins made of approximately 10^7^ copies of a single protein expressed on the surface of the parasite, however, because of antigenic variation this approach failed [35]. Considerable success has been recorded by exploiting non- (or less) variable surface molecules necessary for uptake of nutrients, protein trafficking, endo, and exocytosis, amongst others [36]. These antigens are mostly found in the flagella pocket (FP) and immunization of cattle with antigens located in the flagella pocket showed partial protection [37]. In a mouse model, it showed a 60% success rate, which was overcome by challenges with a higher parasite load (inoculums of 10^3^ parasites or more), indicating that the induced protection conferred boarder-line immunity and was temporal [38]. Several specific invariant surface glycoproteins have been tried as vaccine candidates, among which is transferrin receptor ESAG 6/7. Immunization with sub-cellular antigens, actin and tubulin, involved in cell division and locomotion, have shown varying degrees of protection, with the latter recording 60–80% in an animal model [39]. Unfortunately, the design of the experiment did not permit establishment of the fact that memory of the immune response was stored in memory B cells, and no sound explanation was advanced for antibodies having access to intracellular cytoskeleton protein targets (actin and tubulin). Anti-disease vaccines have been able to alleviate the symptoms of the disease e.g. prior treatment of the host with liposome-based GPI alleviated disease symptoms such as weight loss, anaemia, liver damage, and locomotion impairment, but no memory was stored as these results can be reproduced in B-cell deficient animals [40]. Likewise Congo pain, a cysteine protease, has been assessed as an anti-disease vaccine, but it only reduced anaemia and led to weight gain in the study group of animals, with no significant difference between immunized and non-immunized controls [41].

##### Leishmaniasis

A vaccine against leishmaniasis is scientifically feasible because, historically, it had been observed that individuals who had healed their skin lesions from cutaneous leishmaniasis were protected from further infections [5-7]. This phenomenon was exploited by the Bedouin and some Kurdistani societies to acquire protection from facial lesions later in life by exposing babies’ bottoms to sand fly bites, or by transfer of infectious materials from lesions to uninfected individuals as was done in the Middle East (leishmanization). However, these practices were abandoned by 1990 based on the possibility of developing large uncontrolled skin lesions, exacerbation of skin diseases such as psoriasis, and immunosuppression demonstrated by the poor response of vaccines to diphtheria, pertussis and tetanus triple vaccine [42,43]. Interest was then turned towards killed parasites, and it was shown that vaccination with killed parasite plus CpG adjuvant conferred protection against needle challenge but not against vector transmitted parasites. However, live attenuated parasites were able to confer immunity against transmitted parasites, suggesting that parasite persistence may be necessary for protective immunity pre-munitions [42,43]. Attenuated parasite for vaccination has been achieved by long term *in vitro* culture, selection for temperature sensitivity, chemical mutagenesis, and irradiation [44]. Based on attenuation, vaccination with dihydrofolate reductase or thymidylate synthase (DHFR-TS) knockout parasites led to protection in a mouse model, but not in a monkey model [45]. Deletion of cysteine proteases in *L. major* led to partial protection in an animal model, which was thought to be a result of rapid clearance of self-limited parasites [46,47]. Knockouts of Ipg2 deficient parasites persisted and offered better protection, but, over time, regained their virulent property by an unknown compensatory mechanism [48,49]. SIR2 single knockout strain of *L. infantum* confers protection, but the presence of the second allele of SIR2 raises the probability of reversal to virulence [50]. A non-virulent strain such as the *L. tarentolae* of lizard has shown protection against visceral leishmaniasis in a mouse model [51]. However this approach has the limitation of safety and challenges associated with large-scale production. Sub-unit vaccines are an attractive alternative for leishmania, and amongst the interesting molecules studied are surface expressed glycoprotein leishmanolysin (gp63), which elicited a strong immune response in an animal model but had very little or no T cell response in humans [52]. Parasite surface antigen 2 (PSA-2) involved in invasion by binding to complement receptor 3 has shown protection in its native form but not as a recombinant antigen [53-55]. Leishmania homologue for receptors of activated c Kinase (LACK) has shown protection in BALB/c mice challenged with *L. major*, but the immune response was skewed to detrimental Th2 and did not protect against VL [56-58]. Other antigens have been newly identified. However, the only second-generation vaccine candidate that has been clinically tried is Leish-111f, a chimeric protein of *L. major*, homologue of eukaryotic thiol-specific antioxidant (TSA*)*, *L. major* stress-inducible protein-1 (lmTI1), and *L. braziliensis* elongation and initiation factor (LeiF), which protected mice against *L. major* and *L. amazonensis*, and showed partial protection in an animal model against VL but did not protect dogs in the Phase III trial [59-62]. Human Phase I and II clinical trials have been carried out on Leish 111 f. An improved construct Leish 110f in combination with chemotherapy has been used to reduce the death rate and increase survival probability [63,64]. Presently, research in VL (the worst form of leishmaniasis) is greatly slowed down by the lack of an appropriate animal model for the disease, and our limited understanding of the mechanism of long-lasting protective immunity.

### Disease management

#### Progress and challenges in chemotherapy to combat protozoan diseases

The sector of drug research for diseases of the poor has attracted less attention than other sectors despite their heavy burden. Consequently, only a limited number of pharmaceuticals are currently in use, with a few candidates still in the pipeline (see Tables 2 and 3) [65].

**Table 2:** Limitations and desired product profiles of drugs for malaria, leishmania, Human African Trypanosomiasis, and Chagas disease

**Drugs**	**Limitations**	**Desired profile of new products**
***Malaria***		
Quinine (***Quinine sulphate*, *Quinimax***) (1930)	Compliance, resistance (1960s), safety	Active against resistant strains; oral formulations, with option for parenteral use for patients in coma; use in pediatric formulation; potential combination with other agents; use in pregnancy; cure in three days; stable under tropical conditions; inexpensive.
Chloroquine (***Nivaquine*, *Aralen***) (1945)	Resistance (1950s)	
Primaquine (1948)	Safety, contra-indicated in G6PD deficiency, pregnancy	
Sulphadoxine-pyrimethamine (***Maloxine*, *Fansidar***) (1961)	Resistance (1960s)	
Amodiaquine (***Camoquin***) (1950)	Resistance, safety	
Artemisinins (1994)	Cost, resistance (2008), potential neurotoxicity	
Mefloquine (***Lariam*, *Mephaquine***) (1984)	Resistance (1980s), cost, contra-indicated in known or suspected history of neuropsychiatric disorder	
	Resistance, cost, safety, or recent (<3 weeks) use of Halofantrine	
Halofantrine (1975)	Compliance, resistance potential, contra-indicated in cardiac disease and pregnancy	
Artemether/lumefantrine (***Coartem*, *Mephaquine***) (2001)	Compliance, cost, resistance, GMP, potential neurotoxicity	
Artesunate/amodiaquine (***ASAQ***) (2007)	Compliance, cost, resistance, GMP, safety, contra-indicated in pregnancy	
Atovaquone/proguanil (1999)	Cost, resistance potential	
Tetracycline (1940s), doxycycline (1960s)	Contra-indicated for those aged less than eight years and in pregnancy	
Clindamycin (***Dalacin*, *Lincocin***) (1968)	Efficacy, contra-indicated in severe hepatic or renal impairment; history of gastrointestinal disease, especially colitis	

Adapted from Schiltzer [68], Nwaka and Ridley [75], Nwaka and Hudson [78] and DNDi [79].

**Table 3:** Limitations and desired product profiles of drugs for leishmania, Human African Trypanosomiasis, and Chagas disease

**Drugs**	**Limitations**	**Desired profiles of new products**
***Leishmaniasis***		
Antimonials (1950)	Safety, poor compliance, resistance	Active against resistant strains; oral drug or safe injectable; cure in less than 28 days; pediatric formulation; potential combination with other agents; use in pregnancy; stable under tropical conditions; affordable
Pentamidine (***Lomidine***) (1939)	Safety, poor compliance, resistance	
Amphotericin B (***Fungizone***) (1959)	Safety, poor compliance, resistance	
Liposomal amphotericin B (***AmBisome***) (1990)	Safety, poor compliance, resistance	
Miltefosine (2002)	Safety, poor compliance, resistance	
Sodium Stibogluconate/paromomycin (SSG&PM) (2010)	Contra-indicated in pregnancy	
***Human African Trypanosomiasis***		
Suramin (1920)	Efficacy, injectable	Use against early and late stage disease; active against both major species; parenteral with option for oral use; cure in less than 14 days; pediatric formulation; potential combination with other agents; use in pregnancy; stable under tropical conditions; affordable
Melarsoprol (1949)	Safety, injectable	
Pentamidine (1939)	Resistance, compliance, injectable	
Eflornithine (1991)	Cost, injectable, efficacy	
NECT (Nifurtimox/eflornithine) (2009)	Cost, injectable, compliance	
***Chagas disease***		
Benznidazole (1970)	Activity limited to acute stage of disease, some safety issues	Active against blood and tissue forms of parasite; active in prevention of chronic stage of the disease; pediatric formulation; potential combination with other agents; use in pregnancy; stable under tropical conditions; affordable
Nifurtimox (1974)	Activity limited to acute stage of disease, some safety issues	

Adapted from Adapted from Nwaka and Ridley [75], Nwaka and Hudson [78] and DNDi [79].

##### Malaria

**Figure 2:**
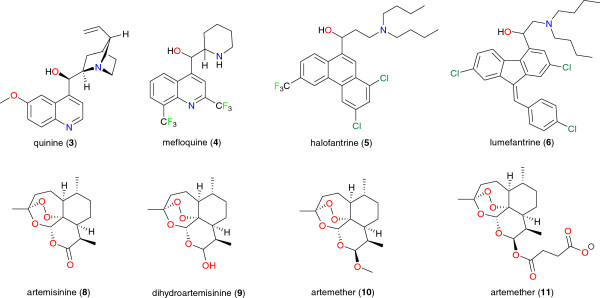
Chemical structures of some antimalarial drugs currently in use.

Relatively low-cost treatment regiments are available against malaria, but the emergence and persistent spread of resistance against all existing therapies have aggravated the disease burden in endemic regions [66,67]. Based on their chemical nature, the currently used antimalarials can be grouped under nine classes (see Figure 2): 4-aminoquinolines, 8-aminoquinolines, amino-alcohols, sulfamines and sulfones, Biguanides, diaminopyrimidine, sesquiterpenes lactones, naphthoquinones, and antibiotics [68]. Despite a large number of antimalarial drugs available, there is no perfect drug; each individual drug or drug combination has its own limitations ranging from poor compliance, side effects, toxicity, or resistance. For several decades, drug resistance has remained the greatest challenge to malaria control, and is one of the obstacles that sapped the dream of seeing malaria eradicated by the 1970s. So far, resistance has been fully established in three of the five *Plasmodium* species responsible for human malaria (*P. falciparum*, *P. vivax*, and *P. malariae*), and this concerns virtually all drug regiments in current use. With this challenge, monotherapies have been strongly discouraged in favor of combination therapies. Several formulations are currently used and contain two or more individual drugs which differ by the targets in the parasite and the half-life time. In order to prevent or delay the emergence of resistance to artemisinin, the most effective drugs for uncomplicated malaria, Artemisinin-based Combination Therapies (ACT) have been strongly recommended by the WHO, and quinolines were selected as the preferred partner drug to artemisinins. The choice of this class of compounds in the formulation of ACTs was justified by the fact that they are long-acting drugs and have different targets from the ones of artemisinins in *Plasmodium*. By 2011, 79 countries had adopted ACTs as the first-line treatment for *P. falciparum.* Consequently, the number of ACT-treatment courses delivered to both public and private sectors globally increased from 11 million in 2005 to 278 million in 2011. A total of 36 out of 45 Sub-Saharan African countries had adopted Intermittent Preventive Treatment (IPT) for pregnant women by December 2011. In 25 of the 36 high-burden countries in the WHO African region, 44% of pregnant women attending antenatal clinics received two doses of IPT in 2011 [2]. This coverage in IPT remains unacceptably low in some 16 countries in the African continent, particularly in Nigeria and DR Congo. In 2012, the WHO recommended a seasonal malaria chemoprevention for children aged 3–59 months, but this new intervention tool is yet to be adopted by individual countries. Sulphadoxine-pyrimethamine (SP), administered either at health facilities or as self-medication, is the most recommended chemotherapy in Cameroon and several other countries in Sub-Saharan Africa. Drug resistance occurs as a phenotype of mutation affecting parasite genome conferring evasion from drug targeting through any of the following mechanisms: drug inactivation or modification, active efflux, and alterations in the primary site of action or metabolic pathway [69,70]. Over time, resistance becomes established in the population, and can be very stable and persisting long after specific drug pressure is removed [69]. Resistance to artemisinins has been detected in four countries in South East Asia: Cambodia, Myanmar, Thailand, and Vietnam. There is an urgent need to expand containment efforts in affected countries, as well as neighboring regions [67]. Numerous factors have been identified to influence drug resistance: i) the intrinsic frequency with which the genetic changes occur; ii) the degree of resistance conferred by the genetic change; iii) the “fitness cost” of the resistance mechanism; iv) the proportion of all transmissible infectious agents exposed to the drug (exposure pressure); v) the number of parasites exposed to the drug; vi) the concentration of the drug to which the parasite is exposed; vii) the pharmacokinetics and pharmacodynamics of the antimalarial medicine; viii) individual (dosing, duration, compliance) and community (quality, availability, distribution) patterns of drug use; ix) the immunity profile of the community and the individual; x) the simultaneous presence of other antimalarial drugs or substances in the blood to which the parasite is not resistant; xi) the transmission intensity [69-71].

Today, in addition to drug resistance, the counterfeiting of pharmaceuticals, especially antimalarials, is also a well-established and alarming public health concern in most of the endemic countries [72-74]. A recent study was carried out by the WHO targeting artemisinins and SP circulating in six selected Sub-Saharan African countries. Out of the 160 samples collected in Cameroon, from both illicit markets and legally established pharmacies or institutions, 37% did not meet the pre-specified internationally acceptable quality criteria. The medicines with the highest failure rates were artesunate-amodiaquine combinations, and up to half of the SP samples failed predominantly in dissolution as well [74]. This situation indicated that more attention is urgently needed both at the local and international levels to ensure the quality of the products circulating. This implies a strict adherence to GCP, GLP, as well as GMP rules and regulations. In addition, more should be invested in pharmacovigilance.

##### Leishmaniasis

**Figure 3:**
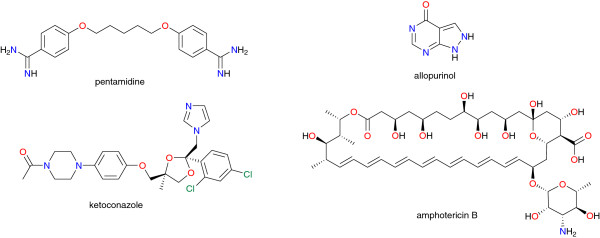
Chemical structures of some antileishmanial drugs currently in use.

A limited number of drugs are available for the treatment of leishmaniasis and these face challenges including limited efficacy for different strains and species, toxicity, affordability in poor communities, and development of drug resistance (see Table 3, Figure 3) [3,75]. Furthermore, these therapies are highly costly thus unaffordable to most concerned, i.e. patients living in low-income remote areas of endemic countries, and they are also subject to drug resistance issues [76-79]. Although no new drugs have been developed recently, a number of clinical trials have been undertaken on a handful of drug candidates, resulting in fruitful outcomes. These include allopurinol, a drug currently used for the treatment of gout. This molecule was shown to inhibit the enzyme hypoxanthine guanine phosphoribosyltransferase (HGPRTase), interfering with protein synthesis in leishmania. Allopurinol is effectively used in veterinary medicine against the canine’s form of leishmaniasis. It is equally under trial for the treatment of Chagas disease in addition to its antileishmanial potential. However, allopurinol was observed to cause hypersensitivity with several adverse effects including chronic kidney disease, hypertension, and higher cholesterol, among others [80,81]. Ketoconazole, an inhibitor of cytochrome P450 designed by Janssen Pharmaceutica, is effectively used for the treatment of candidiasis and other fungal infections. It was recently shown to interfere with sterol synthesis in leishmania leading to the inhibition of growth and cell division in amastigotes. The drug candidate is under clinical trial for use in dogs and in humans in certain Latin American countries [82]. More promising again is miltefosine, an oral medication with anti-leishmanial activity [83].

##### Human African Trypanosomiasis (HAT)

**Figure 4:**
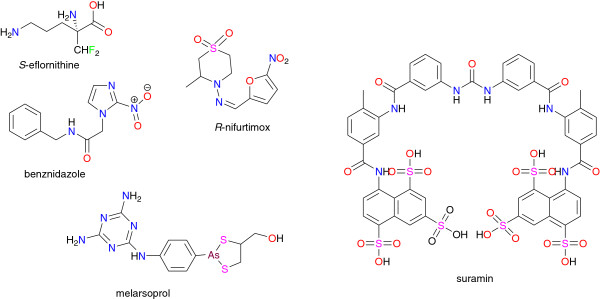
Chemical structures of some antitrypanosomial drugs currently in use.

HAT control is seriously hindered because only a few drugs are available, all of which have significant drawbacks including mandatory parenteral administration, unaffordability, and unacceptable toxicity. Only a handful of drugs—melarsoprol, nifurtimox, and eflornithine (see Figure 4)—are efficacious against cerebral stage 2 disease occurring in the West African type of sleeping sickness [3,77,84]. Drug resistance, especially after melarsoprol treatment reported in several African countries (DR Congo, Sudan, Uganda, and Angola), represents a growing challenge to the control of African trypanosomiasis [3,85]. Enormous efforts are being invested to improve the use of currently registered drugs, including a shortened ten-day course (rather than 21–35 days) of melarsoprol that followed pharmacokinetic studies and a clinical trial with a three-day course of pentamidine. The orally available pro-drug pafuramidine, which was in clinical trials for first stage disease, encountered issues of toxicity [3]. Conclusive results from Phase III evaluation have led to inclusion, in the WHO essential drug list, of nifurtimox in combination with eflornithine to treat HAT. The druggable molecule fexinidazole showed very significant activity from a stage 2 mouse model of HAT, and is currently in Phase I of clinical evaluation by the Drugs for Neglected Diseases Initiative (DNDi) in partnership with Sanofi-Aventis [3].

##### Chagas disease

The goal of a specific treatment against *T. cruzi* infection is to eliminate the parasite from the infected individual and, accordingly, to decrease the probability of developing symptomatic Chagas disease, and hinder parasite transmission [3]. Surprisingly, only two drugs registered more than 40 years ago continue to be used for Chagas disease despite the widespread burden of the disease [3]. Both molecules, nifurtimox and benznidazole, require prolonged treatment (60 days) and have frequent side effects that can lead to discontinuation of treatment. In addition, they are genotoxic, which precludes treatment during pregnancy [3]. The TDR Disease Reference Group on Chagas Disease, Human African Trypanosomiasis and Leishmaniasis suggests that the priorities in Chagas disease research and development (R&D) should be to produce new drugs that provide a shorter treatment course with fewer side effects, and also to devise pediatric formulations. In this regard, some of the most promising approaches are ergosterol biosynthesis inhibitors, such as posaconazole. This drug candidate is under Phase II of clinical trial since October 2010 in Spain [86] and July 2011 in Argentina, sponsored by Merck Sharp & Dohme Corp. [87]. Additionally, DNDi in partnership with the pharmaceutical company Eisai Co., Ltd., is currently conducting a Phase II trial of Benznidazole (E1224), a pro-drug of ravuconazole (E1224). This project started in Bolivia in July 2011 [88].

### Increasing investment in research and development (R&D) targeting vector-borne protozoan diseases

**Figure 5:**
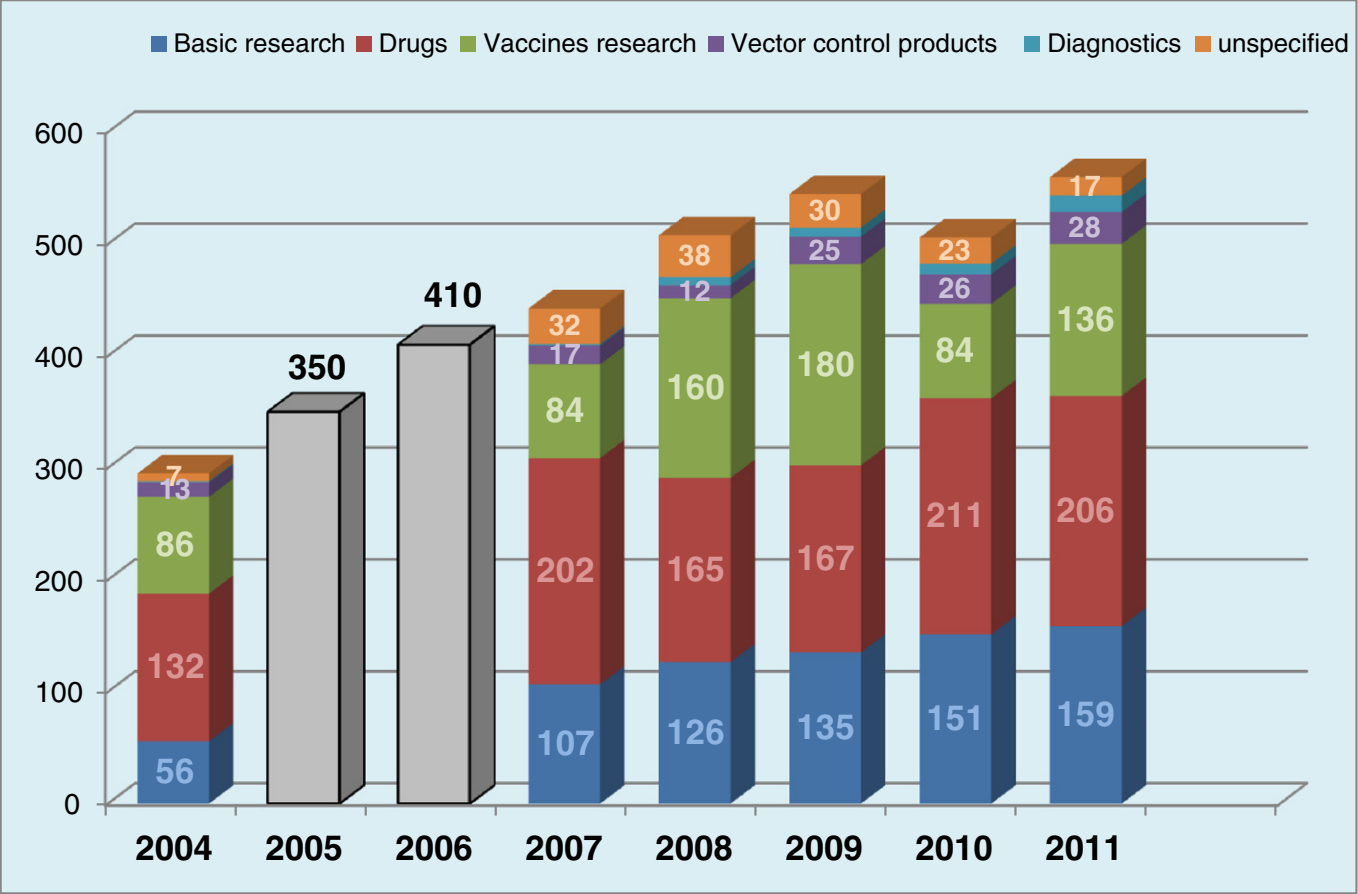
**Distribution of global funding towards drug R&D for protozoan diseases: 2007–2009.** Source: PATH [89], RBM [90], G-FINDER [92]. Distribution data not available for 2005 and 2006.

**Figure 6:**
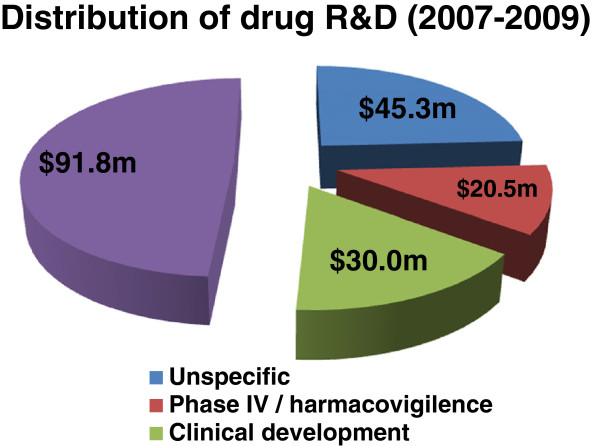
**Distribution of the global funding for R&D targeting protozoan diseases.** PATH [89], RBM [90], G-FINDER [92].

Investment in malaria research and development (R&D) has quadrupled in the past 16 years, from $US121 million in 1993 to $US612 million in 2009 (see Figures 5 and 6). Of these funds, 38% was invested in drug R&D, 28% in vaccines, 23% in basic research, 4% in vector control products, 1% in diagnostics, and the remaining in other related researches. Among the main sponsor organizations worldwide, the Bill & Melinda Gates Foundation and the US National Institutes of Health (NIH) provided a striking half of the global malaria R&D funding in 2007–2009, and were responsible for 85% of the global increase in malaria funding. The Gates Foundation was the single largest funder, providing 30% of global funding in 2009, while the US NIH provided 19%. In the public sector, the USA dominated, providing more than half of all public investment each year, and five times more than any other government [28,89,90]. The action of these funding bodies is fostered by technical support; the coordinating and networking contribution of Contract Research organizations (CROs) such as Medicines for Malaria Venture (MMV), Drugs for Neglected Diseases Initiative (DNDi), and others; and internationally renowned pharmaceuticals companies such as Novartis (pioneer of ACTs), Sanofi-Aventis, and Pfizer among others. For instance, Sanofi has put in place a malaria drug R&D initiative which made significant input into malaria control through ACT formulations, and Novartis recently established the Biomedical Research Institute dedicated to Tropical Diseases, with laboratories based in Singapore. Equally, GlaxoSmithKline (GSK) has set up a R&D initiative targeting major diseases of the developing world. The significant achievements recorded since 2000 can therefore be attributed to the increasing implication of both international and local authorities in the fight against malaria. For instance, 79 of the 104 total endemic countries in 2012 are classified as being in the control phase, while ten are in the pre-elimination phase and ten are in elimination phase. Furthermore, five countries are considered to be in the prevention of the re-introduction phase. Of the 58 malaria-endemic countries with complete data on malaria cases between 2000 and 2011, 50, including nine countries in the African region, are on track to meet the World Health Assembly (WHA) and Roll Back Malaria (RBM) targets to reduce incidence of malaria cases by 75% by 2015. Four other countries are projected to achieve 50–75% reduction. However, three Latin American countries have, instead, witnessed significant increases in malaria cases [8]. At national levels funding had also been consistent for some countries. In general, between 2003 and 2009, 81 of the 108 malaria endemic nations spent their own local resources independently of financial support from the global community for their malaria-control work [91]. Pigott et al. [92] reports that in the period 2006–2010, eight malaria endemic countries (Belize, Costa Rica, Iraq, Malaysia, Panama, Paraguay, Republic of Korea, Saudi Arabia, Turkey) had pursued and sustained their malaria control programs with no international support, and four others (Argentina, Cape Verde, El Salvador, Mexico) received less than $US50,000 cumulative funding from the international community. However, none of these countries are in Africa, and they are all characterized by small populations at risk, a low level of *falciparum* malaria and above-average GDP per capita.

However, since 2007, there has been a steady decline in funding for drug R&D (down to $US49 million, 21%), These trends may be explained, in part, by the maturity of the drug portfolio, with successful registration of several new antimalarials, including artesunate/amodiaquine (2008), artesunate/mefloquine (AS/MQ, 2008) and Coartem® Dispersible pediatric formulation (2009), and submission for registration of Eurartesim™ (2010) and Pyramax® (2010)—as well as the termination of unsuccessful drug candidates, including isoquine (2008) and chlorproguanil-dapsone-artesunate (2009) [28,89-92]. These achievements may have been interpreted as critical steps towards malaria eradiation, underestimating the steady threat of drug resistance. Furthermore, the majority of countries in the African region are control focused, with strategies heavily funded by external donors including vector control and subsidy for existing ACTs. African governments should be more present to provide institutional and financial support, and create an environment conducive to R&D, instead of relying on the lone support from developed countries.

## Conclusions

Despite significant efforts both at the international and local levels in containing the burden of malaria and trypanosomatid infections, growing challenges remain including the difficulties in developing effective vaccines, coupled with the various limitations of existing therapies, the emergence and rapid spreads of resistance against insecticides, and the available drugs. It is crucial to optimize the exploitation of existing facilities through a number of approaches to drug discovery and development. While vaccine research should continue to be supported, interventions in vector control and drugs need special and sustained efforts. Biological tools in vector control look highly promising and the innovation deserves a particular attention. Finally, based on past experiences and the predominant role played by natural products in tropical regions, it is reasonably hoped this leads (notably from medicinal plants) merit special consideration in the development of the next generation of drugs against these diseases. Work should be moved beyond preliminary studies, to include in *vivo* screening, AMEDT, and target identification and validation, which are likely to yield potent new drug candidates (e.g. phytomedicine from multi-potent herbal medicines), for the good of the poor populations suffering the burden of these parasitic infections in Africa, Asia, and Latin America.

## Competing interests

The authors have no commercial or interests in other associations that might pose a conflict of interest.

## Authors’ contributions

DZ and VK conceived the review concept, carried out the literature search, developed the structure for the manuscript, and drafted the paper. RBN, DSN, and FNK participated to organize the draft sections, co-wrote sections of the draft, and edited the overall manuscript. HDM and JCNA participated in the editing of the overall manuscript. All authors read and approved the final version of the manuscript before its submission to IDP.

## Supplementary Material

Additional file 1 Multilingual abstracts in the six official working languages of the United Nations. Click here for file
