# Supplementary material for: Control of malaria and other vector-borne protozoan diseases in the tropics: enduring challenges despite considerable progress and achievements
Source: Infect Dis Poverty. 2014 Jan 8;3:1. doi: 10.1186/2049-9957-3-1 (PMC3895778; doi:10.1186/2049-9957-3-1)
Supplement: Additional file 1 — Multilingual abstracts in the six official working languages of the United Nations. [file 2049-9957-3-1-S1.pdf]

Translation of the abstract into the six official working languages of the United Nations

مكافحة الملاريا وغيرها من أمراض الطفيليات وحيدة الخلية التي تنتقل عن طريق الحشرات في المناطق الحارة : التحديات المستمرة رغم النجاحات والإنجازات الملموسة

دينيس زوفو، ريموند بي نياسا، ديكسون إس نساغا، فيديل نتي كانغ، هنري دي ميريكي، جول كليمنت إن أسوب، وفيكتور كيوت

موجز

تمثل أمراض الطفيليات وحيدة الخلية التي تنتقل عن طريق الحشرات تحدياً خطيراً للصحة العامة، وخاصة في المناطق الحارة التي يساعد بها الفقر مع المناخ الملائم للحشرات في تفاقم المشكلة .جميع الاستراتيجيات المختلفة المستخدمة حالياً لمواجهة هذه الأوبئة غير كافية على الإطلاق. على الرغم من الجهود الهائلة، فإن اللقاحات — التي تمثل السلاح المثالي ضد هذه الأمراض الطفيلية — لا تزال غير كافية من حيث التطوير أو التطبيق، وبالتالي فإن المعالجة الكيميائية، ومكافحة الحشرات الناقلة هي المحاولات الفعالة الوحيدة لتخفيف وطأة المرض في الوقت الحاضر. تواجه هذه الاستراتيجيات صعوبات كبيرة في الوقت الحالي بسبب ظاهرة المناعة المكتسبة ضد العقاقير، مما يؤثر تقريبا على جميع أنواع العقاقير المستخدمة حاليا . الدعم المتزايد مؤخراً من المنظمات والحكومات الدولية لبعض البلدان الموبوءة هو موضع ترحيب كبير، و يجب أن يتم توظيفه على النحو الأمثل في أساليب الأبحاث المتنوعة وتطوير الأدوية للتغلب على عبء هذه الأمراض المنتشرة خاصة الملاريا، وداء الليشمانيات، وداء المثقبيات الأفريقي البشري (مرض النوم)، وداء شاغاس.

Translated from English version into Arabic by Ahmed Ahmed, through

TRANSLATORS
WITHOUT BORDERS

热带地区疟疾和其他媒传原虫病的控制：成就显著，但挑战持久

Denis Zofou, Raymond B. Nyasa, Dickson S. Nsagha, Fidele Ntie-Kang, Henry D. Meriki, Jules Clement N. Assob, and Victor Kuete

摘要

媒传原虫病出现了严重的公共卫生挑战，尤其是在热带地区，贫穷与适合媒介生存的气候同为恶化因素。目前针对这类疾病所采取的每一项防治措施都严重不足。尽管非常努力，但抗寄生虫病最理想的武器—疫苗，还没进行充分的研发和应用。化疗和媒介控制是降低这些疾病负担仅有的有效措施。如今，这两项措施也受到药物抗性的巨大挑战，几乎所有的现行用药方案都受到影响。非常希望有来自国际组织和一些流行国家越来越多的支持，这些支持应该合理地应用到药物研发中，以期降低此类流行病的疾病负担，尤其是疟疾、利什曼病、非洲锥虫病和美洲锥虫病.

Translated from English version into Chinese by Yin Jian-hai, through

CDC CHINESE CENTER FOR DISEASE CONTROL AND PREVENTION

NATIONAL INSTITUTE FOR PARASITIC DISEASES

Contrôle de la malaria et d'autres maladies protozoaires vectorielles dans les tropiques : des défis subsistent malgré des progrès et des succès considérables

Denis Zofou, Raymond B. Nyasa, Dickson S. Nsagha, Fidele Ntie-Kang, Henry D. Meriki, Jules Clement N. Assob, et Victor Kuete

Résumé

Les maladies protozoaires vectorielles représentent un défi de santé publique majeur, surtout dans les tropiques où la pauvreté et les climats favorisant les vecteurs sont des facteurs aggravants. Les stratégies mises en œuvre pour faire face à ces fléaux sont loin d'être parfaites. Malgré d'énormes efforts, les vaccins, qui représentent l'arme idéale contre les maladies parasitaires, ne sont pas encore suffisamment développés ni suffisamment distribués. La chimiothérapie et le contrôle des vecteurs demeurent donc les seuls outils efficaces pour lutter contre la maladie. De nos jours, ces deux stratégies ont un ennemi redoutable: le phénomène de la résistance aux médicaments, qui touche quasiment tous les thérapies actuellement utilisées. Les organisations internationales et les gouvernements de certains pays endémiques se montrent de plus en plus solidaires et leur aide précieuse devrait être utilisée au mieux dans les diverses approches de recherche et de développement de médicaments permettant de lutter contre ces maladies communes, notamment la malaria, la leishmaniose, la trypanosomiase africaine et la maladie de Chagas.

Translated from English version into French by Noelleplat, through

TRANSLATORS
WITHOUT BORDERS

Несмотря на значительные успехи и достижения в борьбе с малярией и другими трансмиссивными протозойными заболеваниями в тропиках, вызовы по-прежнему существуют

Денис Зофу, Реймонд Б. Няса, Диксон С. Нсага, Фидель Нти-Канг, Анри Д. Мерики, Жюль Клемент Н. Ассоб и Виктор Куэтэ

Аннотация

Трансмиссивные протозойные заболевания представляют собой серьезную угрозу для здоровья населения, особенно в тропиках, где отягчающими факторами являются нищета в сочетании с климатическими условиями, благоприятствующими развитию трансмиссивных заболеваний. Разнообразные стратегии, применяющиеся в настоящее время для борьбы с этим бичом населения, являются абсолютно неэффективными. Несмотря на огромные усилия, вакцины, являющиеся идеальным оружием против этих паразитарных заболеваний, еще не разработаны и не внедрены в полном объеме. Таким образом, химиотерапия и борьба с переносчиками инфекции являются единственными действенными способами минимизировать бремя заболевания. Сейчас применение обеих стратегий чрезвычайно затрудняется явлением резистентности к лекарственным средствам, влияющим практически на все схемы приема, которые применяются в настоящее время. В последнее время увеличиваются объемы помощи со стороны международных организаций и правительств некоторых стран с эндемическими заболеваниями. Эту помощь необходимо оптимально использовать в разных методах исследования и разработки лекарственных средств, чтобы избавиться от бремени распространенных заболеваний, особенно малярии, лейшманиоза, африканского трипаносомоза человека и американского трипаносомоза.

Translated from English version into Russian by Vadim Yermolenko, through

TRANSLATORS
WITHOUT BORDERS

Control de la malaria y otras protozoonosis transmitidas por vectores en los trópicos: Desafíos persistentes pese a considerables progresos y logros

Denis Zofou, Raymond B. Nyasa, Dickson S. Nsagha, Fidele Ntie-Kang, Henry D. Meriki, Jules Clement N. Assob, y Victor Kuete

Resumen

Las protozoonosis transmitidas vectorialmente representan un serio desafío para la salud pública, especialmente en los trópicos donde la pobreza, junto a un clima favorable al vector, son los factores agravantes.

Todas y cada una de las estrategias actualmente utilizadas para hacer frente a estas plagas son seriamente inadecuadas. A pesar de los enormes esfuerzos, las vacunas –que representan el arma ideal para combatir estas enfermedades parasitarias- no están aún suficientemente desarrolladas y perfeccionadas. La quimioterapia y el control del vector son, pues, los únicos medios que resultan efectivos para minimizar su morbilidad.

En la actualidad, ambas estrategias están siendo también seriamente afectadas por el fenómeno de resistencia a los fármacos, que afecta prácticamente a todos los utilizados normalmente. El creciente apoyo, en la actualidad, de organizaciones internacionales y gobiernos de algunos de los países endémicos es gratamente bienvenido y debería ser explotado de manera óptima en las diversas aproximaciones a la investigación y desarrollo de fármacos para vencer la morbilidad de estas enfermedades prevalentes, especialmente malaria, leishmaniasis, tripanosomiasis humana africana (HAT) y enfermedad de Chagas.

Translated from English version into Spanish by Psicutrinius, through

TRANSLATORS
WITHOUT BORDERS
